# Step-by-step colpotomy in total laparoscopic hysterectomy: a technique to avoid apical support damage to the upper vagina

**DOI:** 10.4274/jtgga.galenos.2019.2018.0123

**Published:** 2019-05-28

**Authors:** Selim Mısırlıoğlu, Tonguç Arslan, Bülent Urman, Cağatay Taşkıran

**Affiliations:** 1 Clinic of Obstetrics and Gynecology, Koç University Hospital, İstanbul, Turkey; 2 Women’s Health Center, American Hospital, İstanbul, Turkey; 3 Department of Obstetrics and Gynecology, Koç University School of Medicine, İstanbul, Turkey

## Abstract

The purpose of this video article is to demonstrate our colpotomy technique that enables maximal protection of the cervical ring, helps to prevent the ureteral injury by distancing, and avoids shortening of the vagina at total laparoscopic hysterectomy. Step-by-step explanation of the colpotomy technique is presented using educational video setting in university-affiliated private hospital. After the uterine artery transection, a VECTEC surgical uterine manipulator (VECTEC, Hauterive, France) was inserted into the vagina in place of the sharp curette. The plastic rotating blade of uterine manipulator was strongly pushed forward into the anterior vaginal fornix. Colpotomy incision was started from the uppermost middle point of an anterior vagina, and extended to both sides with a monopolar L-hook electrocautery at 40 watts cutting mode. Then the manipulator’s blade was maneuvered into the right lateral fornix, and THUNDERBEAT platform (Olympus Medical Systems Corp, Tokyo, Japan) was chosen as the modality of energy for the transection of the rest of the vagina. At the posterior part of colpotomy, the vaginal wall was cut from the uppermost part of uterosacral ligaments, as well. Finally, the left lateral fornix was cut by the same principles, and colpotomy was completed circumferentially. In conclusion, maximal preservation of paracervical ligaments with this technique preserve the apical support of vagina, and avoids shortening of vaginal length. The technique also minimizes the ureteral injury by distancing.

## Introduction

**Figure 1:**
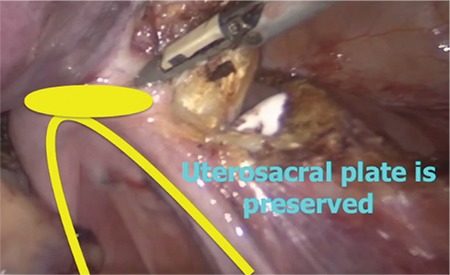
Preservation of uterosacral plate

**Figure 2:**
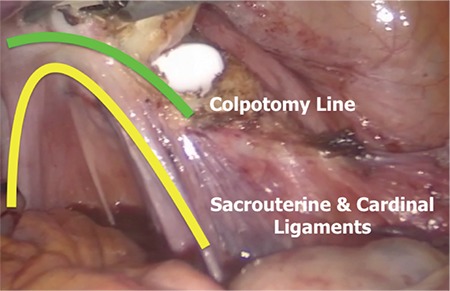
View of colpotomy line

The purpose of this video article is to demonstrate our colpotomy technique that enables maximal protection of the cervical ring, helps to prevent the ureteral injury by distancing, and avoids shortening of the vagina in total laparoscopic hysterectomy. The operation was performed under general anesthesia in the dorsal lithotomy position. The abdominal cavity was insufflated, and a 5-mm primary trocar was placed through the umbilicus. A 30-degree telescope was used for visualization of the peritoneal cavity. A 2.4 mm percutaneous instrument (MINILAP® SYSTEM WITH MINIGRIP® HANDLE) was placed to the upper right quadrant, a 3-mm port to the left lower quadrant, and a 5-mm port to the right lower quadrant. Our hysterectomy technique has been described previously (1, 2). A colpotomy incision was started from the uppermost middle point of anterior vagina, and extended to both sides with a monopolar L-hook electrocautery at 40 watts cutting mode (3). Then the manipulator’s blade was maneuvered into the right lateral fornix, and THUNDERBEAT platform (Olympus Medical Systems Corp, Tokyo, Japan) was chosen as the modality of energy for the transection of the rest of the vagina. After rotating the blade of the manipulator into the lateral fornix, it was pushed forward delineating the connection between the vagina and cervix and then retracted backward to allow space for the THUNDERBEAT. One jaw of the THUNDERBEAT was inserted into the fornix. The vagina was cut from the uppermost part leaving cardinal ligaments maximally on the vaginal side (Figure 1). At the posterior part of colpotomy, the vaginal wall was also cut from the uppermost part of uterosacral ligaments (Figure 2). Finally, the left lateral fornix was using the same principles, and colpotomy was completed circumferentially. By using the manipulator's blade, at the uppermost margin of the vagina, the ureters remained apart from the transection area, the uterosacral and cardinal ligaments were protected, and the vaginal length was preserved maximally (4). After the detachment of the uterus, the specimen was removed vaginally. The vaginal cuff was closed horizontally by using a unidirectional barbed suture (1,5). In our technique, colpotomy starts immediately after the transection of the bilateral uterine artery. In the absence of unnecessary paracervical tissue dissection below this level, the possibility of ureteral injury can be minimized, and the sacrouterine and cardinal ligaments can be maximally preserved (6). Colpotomy is carefully performed above the blade of the uterine manipulator after accessing the anterior vaginal fornix. Transection of cervicovaginal connection from the uppermost part warrants maximal preservation of the cervical ring. A detachment of vagina above the cervical ring can be accomplished via effective uterine manipulation. Stretching tissues by applying enormous pressure on the uterine manipulator is pivotal for exposure of vaginal fornices, which allows easy transection of the uppermost vagina. Maximal preservation of paracervical ligaments with this technique preserves the apical support of the vagina, and avoids shortening of vaginal length. The technique also minimizes ureteral injuries by distancing.
